# The Great East Japan Earthquake reconstruction process in Rikuzentakata City: the trajectory of support activities and local self-sufficiency

**DOI:** 10.3389/fpubh.2026.1806647

**Published:** 2026-05-08

**Authors:** Toshiya Kuchii, Ai Kuroda, Tsunehisa Ito, Yuri Kinoshita, Kanako Sato, Yoshiharu Fukuda

**Affiliations:** 1 Japan Foundation for AIDS Prevention, Tokyo, Japan; 2 Teikyo University Graduate School of Public Health, Itabashi, Japan; 3 Tohoku Seikatsu Bunka University, Sendai, Japan; 4 Aichi Gakuin University, Nisshin, Japan; 5 Kanazawa Gakuin College, Kanazawa, Japan

**Keywords:** men’s cooking class, Rikuzentakata City, self-sufficiency, support activity, the Great East Japan Earthquake reconstruction process

## Abstract

**Objectives:**

The Great East Japan Earthquake of 2011 had devastating effects on Rikuzentakata, leaving the community fragmented and residents isolated. Here, we chronologically report the initiatives and mid- to long-term outcomes of the Red Apron Project’s Men’s Cooking Class, aimed at addressing the isolation of male survivors and fostering community revitalization.

**Methods:**

The trajectory and outcomes of the Men’s Cooking Class were evaluated through focus group interviews with three key informants (*N* = 3) and a chronological review of activities from 2012 to 2020. Data were analyzed using a thematic triangulation process.

**Results:**

The Men’s Cooking Class successfully reduced isolation among male participants, promoted health awareness, and mitigated community divisions by transitioning from temporary housing to community centers. Regular events and door-to-door outreach further strengthened community bonds. The project’s planned termination in 2020, emphasizing self-sufficiency, exemplifies “Responsible Exit.”

**Discussion:**

These findings highlight the importance of consistent support, community engagement, and timely aid withdrawal in rebuilding resilience. This study offers valuable insights for future disaster recovery planning, emphasizing the role of targeted interventions in fostering long-term community recovery.

## 1 Introduction

Rikuzentakata, a city in southern Iwate Prefecture, Japan, was once known for its rich natural resources and an economy centered on the fishing industry. Before the Great East Japan Earthquake on March 11, 2011, the city faced considerable sustainability challenges owing to its aging and declining population. In 2010, Rikuzentakata had approximately 23,000 residents, with 35% classified as older adults (1). The local fishing industry, particularly scallop and wakame seaweed farming, generated annual sales of approximately 1.5 billion yen.

Rikuzentakata was severely affected by the Great East Japan Earthquake. The subsequent tsunami had devastating effects on the urban area, resulting in approximately 2,000 fatalities and affecting one-third of the city’s public employees (2–4). After the disaster, the community encountered numerous challenges in its recovery efforts while managing evacuation centers and navigating widespread confusion.

Immediately after the earthquake, the community developed a sense of solidarity by operating evacuation centers and distributing supplies. For example, in one evacuation center housing approximately 300 evacuees, residents actively participated in food preparation and supply distribution. These collaborative efforts fostered a strong communal bond, even under difficult circumstances. However, new issues emerged as support activities continued, such as the isolation of male survivors and emotional divisions caused by the unequal distribution of supplies. Previous studies have indicated that long-term disaster recovery can span a decade or more, during which the conceptual understanding of disaster risk resilience has continued to evolve (5). During this extended period, men are particularly vulnerable to new social isolation due to the loss of their social roles and traditional gender norms that hinder them from seeking help (6, 7). The “Men’s Shed” model provides a vital theoretical framework to address this vulnerability (8). The fundamental premise of this model is that “women talk face-to-face, while men talk shoulder-to-shoulder.” Research shows that men naturally build peer support and alleviate isolation through shared, purposeful activities, which restores their sense of identity and utility lost after disasters (8). Furthermore, while prolonged external support is essential initially, it can eventually foster over-dependence and hinder the independence of survivors. Therefore, establishing community resilience through “shoulder-to-shoulder” collaborative interventions tailored for men (8) and implementing a “Responsible Exit” strategy are critical challenges in long-term disaster management. The concept of “Responsible Exit” involves planning and implementing the withdrawal of aid in a transparent, respectful, and accountable manner that minimizes harm and ensures the continuity of services (9).

Following the Great East Japan Earthquake, the Ajinomoto Group launched the Red Apron Project in three Tohoku prefectures (Iwate, Miyagi, and Fukushima) to improve the dietary habits of disaster survivors and revitalize communities, and The Ajinomoto Foundation (TAF) succeeded the project (10). In this paper, we chronologically report the trajectory and mid- to long-term outcomes of Red Apron Project’s Men’s Cooking Class, which aimed to support often-isolated men living in temporary housing in Rikuzentakata and ultimately guide them toward sustainable self-reliance.

## 2 Methods

In this study, we employed a qualitative research design using semi-structured interviews to explore the trajectory of the recovery support. From October 2011 to February 2020, the Ajinomoto Group and TAF held collaborative activities with residents approximately once or twice a month. As a post-project evaluation, we selected three key informants using purposive sampling to capture both organizational and beneficiary perspectives. In November 2022, we conducted an interview with one TAF staff member who was deeply involved in the project’s operation and a group interview with two male disaster survivors who were core participants, to summarize the project’s development and gather qualitative data.

To ensure scientific rigor and address researcher reflexivity, the data management and analysis were carefully structured. The analysis was not conducted solely by the principal investigator (YK) to minimize potential bias. Instead, the collected interview data, along with the chronological records of the project’s activities, were systematically analyzed through a triangulation process involving multiple meetings with four researchers. This collaborative thematic analysis ensured the objectivity and validity of the findings regarding the evolution of the support activities and the participants’ transition toward self-reliance.

## 3 Results

The urban area of Rikuzentakata was destroyed by the tsunami caused by the Great East Japan Earthquake, resulting in approximately 2,000 fatalities. One-third of the city’s public employees were killed, leaving the local government inadequately functional. As community soup kitchens were already a part of local culture, residents in unaffected homes began serving meals to evacuees immediately after the disaster. One evacuation center in Rikuzentakata began operating immediately after the earthquake. On the day of the disaster, approximately 300 people gathered at the center, and residents began managing the shelter amidst chaos. During the early stages, the center provided basic meals, such as salted rice balls, which offered an important opportunity for the community to come together.

### 3.1 Trajectory of support activities and transition of participants

Based on the theoretical framework presented in Table 1, the progress of the Men’s Cooking Class can structured as follows.

**Table 1:** Theoretical framework of the four-phase transition process.

Phase	Period	Phase name	Key characteristics and psychological changes
Phase 1	2011–2012	Emergency Support Phase	Focusing on ensuring psychological safety through immediate material aid and the establishment of a “safe haven” for displaced residents
Phase 2	2013–2014	Life Reconstruction Phase	Rebuilding social networks and community ties during the critical transition from temporary shelters to permanent public housing.
Phase 3	2015–2017	Preparation for Self-Reliance	Phasing out prolonged assistance to prevent over-dependence, while ensuring survivors acquire skills for a smooth transition to independence.
Phase 4	2018–2020	Social Integration Phase	Fostering long-term community solidarity and mutual support, leading to the planned termination of support to establish sustainable self-sufficiency.

#### 3.1.1 Phase 1: emergency support phase (2011–2012)

The Ajinomoto Group initiated its program at the Mobilia temporary housing complex, the largest complex in the city. The group organized events 350 days per year and continued to develop activities while assessing the needs of the residents. In 2011, the organizers held events to cook local foods and soba, but the primary attendees were women and older adults. Male survivors became increasingly isolated, and specific efforts were needed to encourage their participation in the community. One supporter recalled: “Men are quite difficult to approach. It was around this time that we began to consider how to attract men. I think we started by wondering if we could hold cooking classes to gather them.” To address the low participation of men, they officially organized an event titled “Men’s Cooking Class” in 2012 and served beer, which led to increased participation from men.

#### 3.1.2 Phase 2: life reconstruction phase (2013–2014)

The earthquake divided residents into those whose homes were destroyed by the tsunami and those whose homes remained intact. By 2013, as rebuilding progressed, further divisions emerged based on financial conditions. Survivors became emotionally divided owing to the unequal distribution of supplies, which hindered cooperation within the community. One participant stated: “We all have walls that separate us. At the time of the disaster, there was a large wall dividing those whose homes were destroyed by the tsunami and those whose homes were not. Naturally, the residents who lost their homes received far more relief supplies.” To address these divisions, the organizers relocated their base from temporary housing to community centers in 2014 and began holding cooking classes for the broader community. The class was renamed “The Tasteful Male Cook,” and matching red aprons were created to promote a sense of togetherness among the participants.

#### 3.1.3 Phase 3: preparation for self-reliance (2015–2017)

During the fourth and fifth years following the disaster, a major issue was ending support for survivors and promoting their self-sufficiency. As prolonged support could hinder the independence of survivors, the group needed to gradually phase out assistance. One staff member noted: “The residents’ reliance on support and provisions is actually a major issue. It is not beneficial for the residents to have their supporters nearby indefinitely.” Throughout this process, they ensured that the residents acquired new skills and became more self-reliant, facilitating a smooth transition from support to independence.

#### 3.1.4 Phase 4: social integration phase (2018–2020)

The cooking classes served as a community hub, providing men with opportunities to connect and feel refreshed. The emphasis was on fostering friendships and mutual support rather than solely on cooking. The class also improved participants’ eating habits. The long-term efforts strengthened community solidarity, and residents found new hope. A participant reflected on the outcomes around 2019: “I felt that Ajinomoto’s 8 years of support to the region was a sign of their passion, and the participants felt the same way.” In 2020, the NPO decided to terminate the project, recognizing that prolonged support would hinder the progress of residents toward self-sufficiency.

The specific trajectory of the interventions and the qualitative changes in the participants across these phases are summarized in Table 2.

**Table 2:** Trajectory of the “Men’s Cooking Class” and participants’ transition in Rikuzentakata.

Year/Phase	Location	Specific events and interventions	Participant changes and social capital
2011–2012/Emergency Support	Temporary housing assembly halls	2011: “Soba-making” experience held. 2012: Official start of the “Men’s Cooking Class,” supported by the Ajinomoto Group (recipes/ingredients) and a local NPO (venue/recruitment).	Male survivors initially tended to be isolated. Cooking provided an opportunity to expand social circles, fostering initial bonding social capital.
2013–2014/Life Reconstruction	District community centers, etc.	Relocated to open community centers to encourage broader participation. Renamed to “The Tasteful Male Cook” and created matching red aprons to strengthen solidarity.	Expanded connections beyond the cooking class to the broader community, developing bridging social capital. Increased health awareness (e.g., salt reduction).
2015–2017/Preparation for Self-Reliance	Same as Phase 2	Gradual reduction of ingredient support and focus on skill acquisition for independent operation.	Shifted from reliance on supporters to acquiring management and cooking skills, fostering self-efficacy.
2018–2020/Self-Reliance and Social Integration	Local community (e.g., festival venues)	Participants actively provided cooking class menus at temporary housing festivals. Feb 2020: Conclusion of the 8-year collaborative activities. June 2020: Planned dissolution of the local NPO, marking a responsible exit (termination of support).	Improved self-efficacy and confidence through positive community feedback. Complete transition from passive recipients to active community contributors.
2022/Post-Project Evaluation	–	Nov 2022: Longitudinal assessment of the project’s impact through interviews.	Confirmed sustained self-reliance; former participants continued to maintain community bonds without external TAF support.

## 4 Discussion

### 4.1 Overview of the study findings

#### 4.1.1 Consistency with existing surveys

The findings of this study, which highlight the importance of solidarity among residents and community revitalization, align with previous efforts to promote soup kitchens and community participation (11–14). Furthermore, previous research conducted after the Great East Japan Earthquake has demonstrated that participation in cooking classes serves as a significant motivator for social interaction and contributes to improved dietary variety among survivors (15).

#### 4.1.2 Social isolation of men post-disaster and its factors

The initial primary objective of the project was to prevent the isolation of male disaster survivors. According to Kotozaki et al. following the Great East Japan Earthquake, men were at a higher risk of falling into new social isolation due to the loss of their homes and sudden environmental changes than women, with the loss of social roles, such as through unemployment, directly linked to isolation (6). Similarly, research in other disaster contexts, such as the floods in Bangladesh, has shown that traditional gender norms dictating that “men should not show weakness” hinder men from seeking support or expressing their emotions post-disaster, ultimately leading to social withdrawal (7). Owing to these factors, there was a strong need for an approach specifically tailored to men during the emergency support phase.

#### 4.1.3 Building social capital and the “men’s cooking class”

The “Men’s Cooking Class” was not merely about cooking instructions, but an intervention that considered the psychological characteristics of men. Recent evidence suggests that higher cooking skills are positively associated with richer social relationships and increased social capital among older Japanese adults (16). As Golding illustrates in the research on the “Men’s Shed” movement, men tend to prefer communicating “shoulder-to-shoulder” through shared tasks rather than conversing “face-to-face” (8). The collaborative work of cooking provided a safe space where men could express themselves without pressure, thereby successfully building bonding social capital among the participants. Moreover, when divisions arose among the survivors owing to differences in the progress of reconstruction, shifting the activity base from the largest temporary housing complex to an open local community center was a critical turning point. This relocation of the base facilitated the formation of bridging social capital, connecting different groups across the community, which is a decisive factor for long-term disaster resilience (17, 18).

#### 4.1.4 Importance of “Responsible Exit” for self-reliance

A major aspect of the project is the termination of support (phase-out) at an appropriate time, transitioning residents from dependence on external aid to self-reliance. Bahati et al. warn of the risks that poorly planned withdrawals of humanitarian aid can have harmful effects on communities, such as dependence and system collapse, and define “Responsible Exit” as a transparent and accountable withdrawal process (9). In this project, the survivors’ transitioned from “passive recipients of support” to “active contributors” who provide meals to their community, and the achievement of a planned termination of support exemplifies “Responsible Exit.”

#### 4.1.5 Study limitations

This study had several limitations. First, as a qualitative study focusing on a specific project in Rikuzentakata City, the findings may not be directly generalizable to other disaster-affected regions with different cultural and social backgrounds. Second, the small number of key informants for post-project evaluation may not capture the full diversity of participant experiences. However, the triangulation of interview data with 8 years of longitudinal activity records ensures a high level of descriptive validity regarding the reconstruction process and the long-term outcomes of the project.

## 5 Conclusion

The trajectory of this 8-year initiative demonstrates that effective long-term disaster recovery extends beyond immediate material relief, requiring targeted psychosocial interventions and a strategic transition toward community self-sufficiency. By addressing the unique vulnerabilities of male survivors through shared, purpose-driven activities, the intervention successfully fostered both bonding and bridging social capital, mitigating isolation and community fragmentation. Furthermore, the planned phase-out of institutional support exemplifies “Responsible Exit.” This approach prevented over-dependence, empowering survivors to transition from passive aid recipients to active community contributors. Ultimately, this study provides a vital framework for future disaster management, highlighting that the timely withdrawal of aid, coupled with local empowerment, is essential for building sustainable community resilience.

## Data Availability

The datasets presented in this article are not readily available because they contain sensitive information regarding disaster victims. Requests to access the datasets should be directed to tkuchii@gmail.com.
